# The trend of feminization of doctors’ workforce in Oman: is it a phenomenon that could rouse the health system?

**DOI:** 10.1186/s12960-018-0283-y

**Published:** 2018-04-27

**Authors:** Nazar A. Mohamed, Nadia Noor Abdulhadi, Abdullah A. Al-Maniri, Nahida R. Al-Lawati, Ahmed M. Al-Qasmi

**Affiliations:** 1 Ministry of Health, Muscat, Sultanate of Oman; 2 Oman Medical Specialty Board, Al Khoudh, Sultanate of Oman

**Keywords:** Feminization, Phenomenon, Doctors, Health workforce, Oman

## Abstract

**Background:**

Participation of women in the medical profession over several countries worldwide was increased over the past decades. This paper is a part of ongoing studies aiming at addressing the issue of health workforce feminization among doctors in the Sultanate of Oman as well as exploring the health system readiness in dealing with this phenomenon.

**Methods:**

Literature in addition to reports and records of the Ministry of Health, Oman (MoH), Sultan Qaboos University (SQU) and Oman Medical Specialty Board were reviewed regarding the gender of the doctors and the medical students.

**Results:**

Findings regarding the medical students at the SQU showed higher number of females compared to males (64% females in 2015 compared to 54% in 2009). A similar trend was observed regarding the postgraduates as 61.5% of the graduated residents doctors were females.

As for active workforce, the MoH 2015 report revealed that female doctors represent 42% of the total doctors compared to 27% in 1990. It increased 4% from 1990 to 2000, doubled to 8% from 2000 to 2010. The proportion of specialized female doctors reached 31% in 2015 compared to 21% in 1990. There were also gender variations among specialities. The proportion of female general practitioners reached 50% in 2015 compared to 30% in 1990 (4% increase every 5 years).

**Conclusions:**

The feminization phenomenon in Oman is increasing and requires more attention in order to assess the health system readiness of meeting the needs and accommodating the females as the main care providers. The trend is expected to have important consequences on future planning, given that women doctors differ from men in how they participate in the workforce. It may also potentially contribute to a shortage in supply due to difference in preferences and consequently affect the skill-mix and productivity. The cultural, social context and dimensions need to be explored and feasible options to be provided for better planning.

## Background

It has been observed over the past decades and intensified over the past few years in many countries over the globe that there are consistent trends of increased participation of females in the medical profession (the so-called feminization of medicine) and that the profession of medicine is not dominated by males as before [1]. The notion of feminization of a profession signifies a variety of meanings. In much of the literature, a profession is feminized when women constitute the majority of its practitioners [2]. However, some authors identified other meanings: those who recognize certain attributes as uniquely feminine (such as empathy, relatedness, nurturance and collectiveness are recognized, valued and expressed in the performance of professional tasks and functions) regard the profession as feminized. Then there is the feminist premise that a profession becomes feminized not by stereotypic attribution of gender qualities, but when its practice and substantive rules adapt and change in such a manner that women who enter the profession do not have to conform to a male model of what it means to be a professional [2, 3]. This paper considers feminization as the increased participation of women in the profession.

Females now comprise a majority or near-majority of medical students and predominate in certain specialities in many high-income countries [4]. For example, in the United States of America, 48% of medical students were women in 2013–2014, up from just 7% in 1965–1966 and women now account for more than half of graduate trainees in seven specialities. Similar trends are found across the United Kingdom, Canada, Australia and various European countries [4].

The proportion of female doctors in countries of the Organization for Economic Co-operation and Development (OECD) grew by nearly 16% between 1990 and 2013 [5]. In 2013, 45% of doctors on average across OECD countries were females, up from 38% in 2000 and 29% in 1990. At least half of all doctors now are females in 10 countries, ranging from 50% such as in Spain and Netherland, to 74% as in Estonia [5]. By contrast, about one-in-five doctors in Japan and Korea were females in 2013 [5]. Females are representing an increasing proportion of the physician workforce in low- and middle-income countries. However, the phenomenon is generally less well studied [4].

Concerning the Arab and Gulf countries, there were few published data found regarding feminization of medical workforce. A study in Sudan has shown that the proportion of female medical students has increased up to 69.5% forming almost 64.7% of the total graduates in the period 2000–2009 [6]. While for the total health workforce, there was slight dominance of females (51%) but with increasing trend as illustrated in the national human resources for health strategic plan 2012–2016 [7].

The Health Workforce 2030 Global Strategy [8] has reaffirmed that health workforce will be critical to achieving health and wider sustainable development goals (SDGs). The health targets under consideration in the SDGs include a renewed focus on equity and universal health coverage [8]. This can be attained through substantive and strategic investments in health workforce for both males and females. Improving health services coverage and health outcomes are much dependent on the availability, accessibility, acceptability and quality human resources for health [8, 9].

The Sultanate of Oman is located in the south eastern corner of the Arabian Peninsula. Administratively, the country is divided into 11 governorates and 61 districts, locally known as (Wilayats) distributed among these governorates. The population size in 2015 was 4 159 102 of whom 43.6% were non-Omani [10].

Oman has achieved universal health coverage through dramatic transformation in its health care system over a remarkably short span of time [11]. The country is being recognized internationally as one of the few countries with successful experience in health development. For instance, the World Health Organization (WHO), in its first-ever comparative analysis of health systems in 2000, ranked Oman first among 191 countries for its overall performance on the level of health [12]. In 2010, the United Nation Development Programme (UNDP) identified top mover countries relative to the starting point in 1970s regarding health development and ranked Oman first among 135 countries worldwide as the most improved nation during the preceding 40 years [13].

A remarkable success has been achieved in evolving policies and plans for controlling and eradicating major communicable diseases [10]. The health indicators in 2015 showed a remarkable reduction in childhood mortality. For instance, the under-five and infant mortality became 11.4 and 9.5 per 1000 live birth compared to 86 and 64 per 1000 life birth in 1980 [10]. Furthermore, the life expectancy at birth reached 76.4 years in 2015 compared to 57.5 years in 1980 [10].

As for the health workforce, the density of doctors and nurses per population in Oman has increased remarkably. As for doctors, it reached 21.4 per 10 000 population in 2015 compared to 5.1 in 1980, while for the nurses, it reached 46.3 per 10 000 population compared to 10.8 in 1980 [10]. However, both densities are still below the OECD average which is 33 doctors per 10 000 population and 91 nurses per 10 000 population in 2015) [5].

Oman continued to develop its educational infrastructure and began to produce as much workforce as possible, in order to meet health care demands and achieve workforce self-reliance.

Different initiatives with a beneficial impact on the workforce development were introduced in the past decades such as the regionalization of health professions’ training institutes, active collaboration with universities and overseas speciality boards, qualitative improvement of the education system and the development of a strong continuing professional development system [14]. The workforce management system treats both males and females health professions on equal footing and there is no discrimination in terms of recruitment, deployment, remuneration, and professional development.

Notably, the health system in Oman is facing a major epidemiologic transition whereby the non-communicable diseases (NCDs) manifested as real burden that will strain the health system if not adequately addressed [11]. The health system in Oman recognized that having the best talented, motivated and competent health care professionals are very critical in addressing the system challenges, tackling the burden of diseases and sustaining universal health coverage through provision of quality health care services [15].

Since the year 2012, the Ministry of Health in Oman has embarked on a vigorous process of modernizing the health system aiming at improving the quality of care and sustaining the health gains. The process yields the development of long-term strategic document “Health Vision 2050” which resembles a landmark in health system development that encompassed almost all issues related to health [11].

The present paper is a part of ongoing studies aiming at addressing the issue of health workforce feminization among doctors in the Sultanate of Oman as well as exploring the health system readiness in dealing with this phenomenon based on Oman Health Vision 2050, Health Workforce 2030 Global Strategy and SDGs and Universal Health Coverage. The studies will be undertaken one by one under subtitles to the main title mentioned in the paragraph above.

The current study is specifically aiming at studying the trend of feminization among medical doctors’ workforce in the Sultanate of Oman and their distribution at work places according to their sexes among the different specialities.

## Methods

The researchers conducted a review of the literature to study the global trend of female’s participation in the medical profession. A literature search by the authors was conducted for publications on feminization of medical staff over the world and in the Gulf Council Cooperation (GCC) region using MEDLINE®. MEDLINE® searches were carried out via PubMed® in the month of July 2017. The World Health Organization (WHO) reports were also included. Two search strategies were adopted one based on the use of appropriate Medical Subjects’ Headings (MeSH) and the second based on text words (free-text searching) for maximal retrieval of relevant articles. The following search terms were used alone or in combination in both strategies: doctors, female doctor, women doctors, men doctors, feminization of medical workforce, health care providers, medical workforce, feminization among medical students, Arab world and Oman. The reference lists from retrieved articles and their citing articles (identified through Scopus®) were scanned to identify additional relevant papers. Basic and clinical research papers, systematic reviews and meta-analyses related to gender of doctors and other medical staffs were included. Non-original publications including reviews, case reports, commentaries, letters and editorials were excluded. Only articles written in English were included in the study. The former cut-off (January 1990 to December 2016) was set to allow for maximum capture of published articles from GCC countries. A 1 year was considered a reasonable lag-time for any submitted manuscripts to be published in full-text.

Furthermore, a review of reports and records regarding the human resources and gender of the health care workers, specifically the doctors, and the medical students in Oman was performed. These data and information were retrieved from the annual statistics of the Ministry of Health (MoH) which is the main provider of health care in the Sultanate of Oman, the Oman Medical Specialty Board (OMSB) which was established in 2005 and mandated to spearhead the development of postgraduate residency programmes in Oman, and Sultan Qaboos University (SQU) which is the sole government institution that graduates medical doctors. Data of MoH were obtained on 5-year interval from 1990 till 2015, while for OMSB since it is established in 2005 whenever possible. This was followed by trend analysis of feminization of health workforce. The data were entered into excel sheet, and descriptive analysis was performed. SPSS Version 19 (IBM) was also used.

## Results

### Feminization of medical students in Oman

The findings regarding feminization of medical students in Oman from the period of 2009 to 2015 showed that the number of female medical students was higher than that of male medical students in SQU. For instance, 64% of the enrolled medical students in 2015 where females compared to 54% in 2009 as shown in Table 1.

**Table 1:** The number and percentage of males and females students in SQU during 2009 to 2015

Year	Enrolled students males		Enrolled students females		Total
	No.	%	No.	%	
2009	61	46	72	54	133
2010	59	37	102	63	161
2011	64	36	116	64	180
2012	54	33	112	67	166
2013	56	36	100	64	156
2014	56	36	99	64	155
2015	55	36	98	64	153

### Feminization among residency training programme doctors

**Fig. 1:**
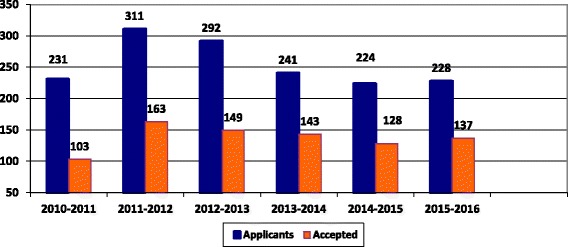
The number of applicants and accepted doctors to OMSB out of the total applicants per year since 2010 to 2016

A similar trend has been observed regarding the residency training programme doctors. The training programmes for the resident doctors are organized by the OMBS. The average intake of OMSB is 150 residents per year in the different speciality programmes. Figure 1 shows the number of accepted residents out of the total applicants per year since 2010 to 2016.

**Fig. 2:**
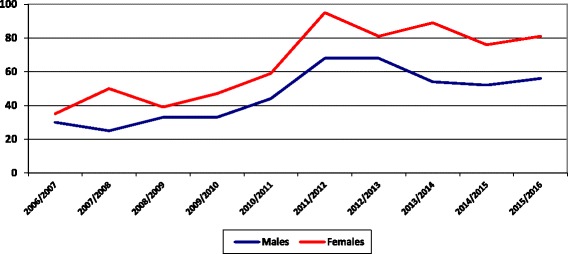
The number of accepted residents in the college of medicine in SQU per gender per year from 2006 to 2016

The analysis by gender since the academic year 2006/2007 to 2015/2016 showed the dominance of females alongside a rising trend as shown in Fig. 2.

### Gender of applicants to the OMSB programme versus choice of speciality of medical field

It has been noticed that female doctors comprised 62.5% on average out of the total pool of applicants with differences in preferences among specialities. For instance, the higher number of applicants for internal medicine, family medicine, paediatrics and dermatology were female doctors, while the applicants for orthopaedics and oral and maxillofacial surgery was higher among male doctors. Details are shown in Table 2.

**Table 2:** Number of applicants in different specialities of medicine from 2010 to 2017

Programmes	Number of applicants per gender per year														Total		
	2010–2011		2011–2012		2012–2013		2013–2014		2014–2015		2015–2016		2016–2017				
	M	F	M	F	M	F	M	F	M	F	M	F	M	F	M	F	Total
Anaesthesia	7	2	1	5	2	2	4	2	1	2	4	6	0	1	19	20	39
Biochemistry	1	0	0	0	1	6	0	0	0	0	0	0	1	4	3	10	13
Dermatology	2	19	5	19	8	26	9	7	1	8	0	0	2	14	27	93	120
Emergency medicine	15	5	13	8	9	10	6	12	6	4	4	9	6	8	59	56	115
E.N.T.	2	3	2	3	2	3	3	9	3	5	4	4	2	10	18	37	55
Family medicine	11	25	22	40	21	38	13	31	24	46	22	45	6	42	119	*267*	386
General surgery	9	2	21	7	12	7	11	11	6	3	7	6	8	11	74	47	121
Haematology	0	0	1	13	1	3	0	3	1	2	2	2	1	9	6	32	38
Histopathology	0	10	0	0	2	1	0	0	4	9	1	8	0	10	7	38	45
Internal medicine	6	3	10	10	14	13	11	16	14	13	13	13	10	9	78	**77**	155
Medical microbiology	3	1	0	0	4	4	0	0	0	3	0	3	1	11	8	22	30
Obst and Gyne	0	11	0	13	0	5	0	7	0	7	0	7	0	5	0	*55*	55
Ophthalmology	5	8	8	15	6	13	6	9	4	3	4	5	1	5	34	*58*	92
Orthopaedics	0	0	15	3	20	0	13	1	12	1	5	0	5	1	*70*	6	76
Oral and maxillofacial surgery (OMFS)	0	0	0	2	4	0	2	0	2	2	4	1	3	3	15	8	23
Paediatrics	12	18	8	24	5	30	12	24	10	12	9	20	9	22	65	*150*	215
Psychiatry	8	12	3	12	4	3	3	7	3	5	2	5	7	7	30	51	81
Radiology	13	18	7	21	5	8	4	5	1	7	3	10	3	23	36	92	128
Sub-total	94	137	116	195	120	172	97	144	92	132	84	144	65	195	668	1119	
Total	231		311		292		241		224		228		260		1787		

**Fig. 3:**
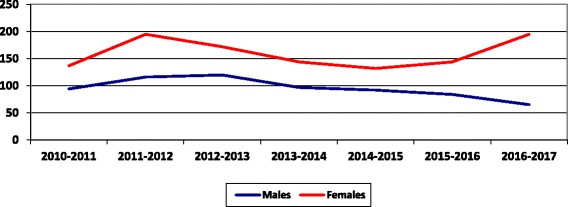
The number of applicants to OMSB per gender per year since 2010 to 2017

The gender distribution of the pool of applicants per year is shown in Fig. 3. The percentage of female doctors’ applicants increased from 59.3% in 2010/2011 to 63.2% in 2015/2016 and it reached 75% in 2016/2017, while the number of male applicants is steadily declining.

### Accepted applicants by OMSB by gender and speciality

**Fig. 4:**
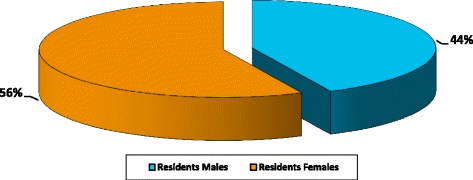
The number of accepted residents by OMSB per gender

**Fig. 5:**
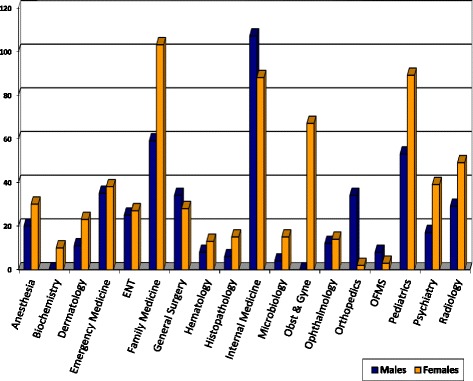
Distribution of OMSB residents in different specialities versus gender

The same trend was observed among residents who are currently enrolled in the various OMSB speciality programmes as shown in Table 3. The gender distribution for the accepted doctors is 56% females and 44% males as shown in Fig. 4, while Fig. 5 shows the distribution of residents by gender in different specialities.

**Table 3:** Number and percentages of accepted residents by OMSB per gender per speciality

Speciality	Frequency	Percentage	Male	Female
Anaesthesia	12	6.9	3	9
Dermatology	3	1.7	2	1
Emergency medicine	4	2.3	0	4
ENT	6	3.4	4	2
Family medicine	37	21.3	13	24
General surgery	2	1.1	2	0
Haematology	8	4.6	4	4
Histopathology	1	0.6	1	0
Internal Medicine	39	22.4	21	18
Microbiology	3	1.7	0	3
OBS and GYN	11	6.3	0	11
Ophthalmology	4	2.3	3	1
Oral and maxillofacial surgery	3	1.7	2	1
Orthopaedics	10	5.7	10	0
Paediatrics	16	9.2	4	12
Psychiatry	9	5.2	4	5
Radiology	6	3.4	4	2
Total	174	100.0	77 (44%)	97 (56%)

As illustrated in Fig. 5, the specialities of family medicine, paediatrics, internal medicine, and obstetrics and gynaecology are highly occupied by female doctors while the other specialities are almost equally occupied by male and female doctors with no great variation.

### Distribution of graduated residents by gender in different specialities

Table 4 illustrates the distribution of graduated residents by gender from 2006/2007 until 2014/2015. It is also shown that female doctors constitute 61.5% of the total OMSB graduated residents. In addition, 8 out of the 17 specialities demonstrated in the table were dominated by female doctors.

**Table 4:** Distribution of graduated residents by gender in different specialities

Programme	2006/2007–2009/2010		2010/2011–2014/2015		Total (2006/2007–2014/2015)	
	M	F	M	F	M	F
Anaesthesia	0	2	8	6	8	8
Paediatrics	8	24	18	32	26	56
Dermatology	8	10	4	10	12	20
Emergency medicine	5	6	17	13	22	19
ENT	3	2	7	4	10	6
Family medicine	13	36	22	36	35	72
General surgery	2	2	8	5	10	7
Internal medicine	10	7	30	16	39	23
Microbiology	1	2	0	7	1	9
Obst and Gyne	0	2	0	14	0	16
Psychiatry	0	7	8	20	8	27
Haematology	0	2	4	1	4	3
Histopathology	1	2	1	9	2	11
Radiology	2	3	8	13	10	16
Biochemistry	0	0	0	6	0	6
OMFS	0	0	1	1	1	1
Ophthalmology	–	–	1	3	1	3
Total	53 (33%)	107 (67%)	137 (41.1%)	196 (58.9%)	190 (38.5%)	303 (61.5%)

### Feminization among medical doctors within the Ministry of Health in Oman

**Fig. 6:**
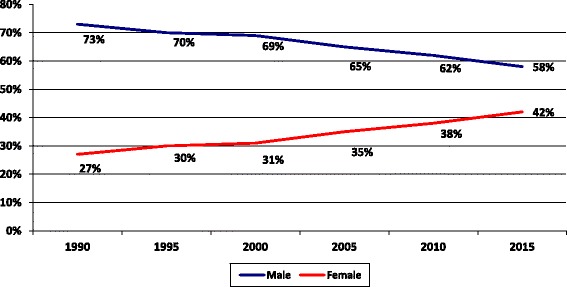
Feminization among medical doctors within the Ministry of Health in Oman

While the proportion of the female medical students in SQU and in female doctors in OMSB has increased over the last years, the same happened in the active workforce. The Ministry of Health (MoH) 2015 annual report revealed that female doctors represented 42% of the total doctors’ workforce compared to 27% in 1990 as shown in Fig. 6.

### Distribution of doctors’ workforce in the Ministry of Health

**Fig. 7:**
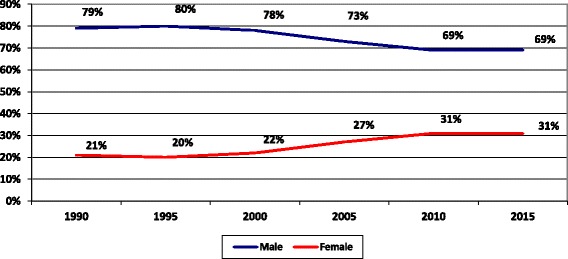
Distribution of specialists and consultant doctors by gender in the MoH

As for the specialists and consultants workforce in the MoH, the proportion of female doctors has reached 31% of the total specialists and consultants in 2015 compared to 21% in 1990 as illustrated in Fig. 7. Also, the percentage of female specialists and consultants working in senior management posts has increased steadily from 0% in 1990 to 30% in 2013.

### Distribution of MoH specialists and consultants

As shown in Table 5, female specialists and consultants were more in obstetrics and gynaecology, family and community medicine, biochemistry and dermatology. While in the contrary, they were below 10% in specialities like general surgery, orthopaedics, ENT, cardiothoracic surgery, gastroenterology, neurosurgery, nephrology and cardiology. This variation is comparable with the OMSB residents.

**Table 5:** Distribution of MOH specialists and consultants per gender and speciality, in the year 2015

Speciality	Male	Female	Total	Speciality	Male	Female	Total
General surgery	191	7	198	Dermatology	27	30	57
Orthopedics	122	3	125	Psychiatry	41	12	53
Urology	15	1	16	Oncology	19	13	32
Cardiothoracic surgery	25	0	25	General pediatrics	160	73	233
Plastic surgery/burns	31	0	31	Neonatology	13	2	15
Neurosurgery	19	1	20	Obst. and gynecology	0	222	222
Pediatric surgery	33	9	42	Pathology/haematology	17	20	37
Anesthesiology	206	40	246	Biochemistry	2	6	8
Ophthalmology	97	31	128	Radiology/radio-diagnosis	69	27	96
ENT	85	9	94	Microbiology	11	13	24
Internal medicine	183	41	224	Family and com. health	28	38	66
Cardiology	54	4	58	Public health	16	19	35
Nephrology	57	4	61	Epidemiology	20	3	23
Gastroenterology	10	1	11	Hospital administration	16	1	17

**Fig. 8:**
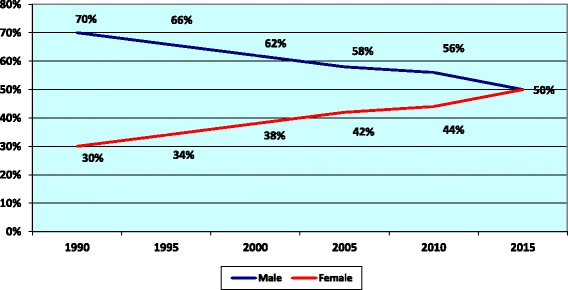
Distribution of MoH general practitioners by gender

While for the general practitioners (GPs) in the MoH, the proportion of females has reached 50% of the total GPs in 2015 compared to 30% in 1990 while the number of male GPs was at higher level to the female GPs before declining to 50% in 2015 as illustrated in Fig. 8.

## Discussion

It is evident from the findings in this study that the feminization of medical doctors in Oman is steadily rising. The percentage of female doctors in MoH has increased by 4% from 1990 to 2000, while it doubled (8%) from 2000 to 2010, and for the GPs particularly, the rise was nearly 4% every 5 years interval. In general, the number of female medical students in Oman is higher than that of the male medical students and the number of applicants and accepted postgraduate doctors for the speciality training programmes was higher among women doctors than men doctors.

The same trend has been observed in many countries. In high-income countries, the number of women entering medical education is superior to that of men [16]. In the Netherlands in 2008, 65% of medical students were females, while in the United States of America in 2007, 49% of medical students are females, compared to only 13% female medical students in 1970 [17]. Some researchers have argued that the roots of the phenomenon are in the changing features of the profession that make it no longer attractive to males as before [2, 18], while there has been greater access to medical training for female students [18]. The medicine graduation courses already had majority of females in several countries, such as England, Ireland and Norway in the 1990s [19]. In several industrialized countries, the proportion of primary care doctors who are females has nearly doubled over the last 30 years [20]. In Canada, 60% of family practice trainees are females and the number of female doctors increased by nearly 23% between 2007 and 2011 compared to a 9% increase in the number of male doctors during the same period of time. Furthermore, in Switzerland, the proportion of female physicians has doubled over the past decades as it increased from 17% in 1980s to 36% in 2010 [21]. A study in Brazil showed that since 2009, females constituted higher numbers than males among the new registered doctors, although males still prevail 60% in the active physician population in the group aged less than 29 years old [19].

The MOH statistics in Oman revealed that the proportion of female doctors among the senior hospital administration was less than 6%. The same was observed in Switzerland whereby the proportion of females among the senior medical staff in hospitals was as less than 10% [21].

According to the OECD, changes in the extent of female participation can have important consequences for the planning of the supply of health care human resources, given that female health care workers tend to differ from males in how they participate in the workforce [22]. In Oman, evidence is required to be made available on whether female doctors differ from male counterparts in terms of the soft skills that males and females bring into a work environment.

As shown in this study and beyond the feminization trend in Oman, there are still marked difference in the preferences among medical specialities both in postgraduates and active doctors workforce, i.e. some specialities were preferred and dominant by females, e.g. family medicine, paediatrics, biochemistry, psychiatry and microbiology, in addition to the obstetrics and gynaecology. On the contrary, orthopaedics and oral and maxillofacial surgery (OFMS) were preferred by males. A study to examine the speciality career choice of applicants to postgraduate training programmes in the United Arab Emirates noted statistically significant differences between gender in career preferences with more females preferring family medicine, paediatrics, dermatology and obstetrics, while the male preferred general surgery, radiology, emergency medicine and urology [23]. Also, literature search on gender differences in medical students’ speciality preferences showed that surgery is predominantly preferred by males and gynaecology, paediatrics and general practice by females [24]. In the context of Oman, an in-depth study is required to examine the preferences among medical specialities.

These variations may potentially contribute to a shortage in service supply due to difference in preferences. Specialities with very low rates of female participation may experience critical shortages in the future. In addition, the uneven distribution across medical specialities has its ramification on the work-related conditions such as skill-mix imbalance, types of contracts, increased risk of stress-related suffering, burnout and productivity [20, 21]. In Scotland, there have been concerns that such a change may lead to increased part-time working and subsequently to a fall in available general practice manpower despite an apparently rising overall number of general practitioners [25].

The feminization of the medical workforce is expected to become more important and could rouse an alarm to the health system in Oman if not well prepared to cope with this phenomenon in the future. Some countries like Sudan has dedicated a special strategy that focused on developing a female-friendly policy for jobs in the underserved areas as sated in the national human resources for health strategic plan 2012–2016 [7].

## Conclusions

The number and percentage of female medical workforce in Oman is progressively increasing. Although it is not an inconvenient situation, however, it might be if not tackled appropriately. The authors are emphasizing that the feminization phenomenon of medical workforce in Oman requires more attention in order to assess the health system capability and readiness of meeting the needs and accommodating the females as the main care providers.

Evidence is required to be made available for the decision makers in order to measure the implications of the feminization of the health workforce in a country like Oman, bearing in mind the cultural, social context and fabric, and explore the options to provide adequately and timely interventions whenever required. It is also important to study and compare the overall career duration, working patterns and conditions, engagement in public and private sectors, practice style, as well as level of productivity among male and female physicians, and whether there is a need to adjust the health human resources modeling and planning, public health, patient care and management policies. The coming studies of this series will cover most of the areas mentioned above.

## Abbreviations

GPs: General practitioners
MoH: Ministry of Health
NCDs: Non-communicable diseases
OECD: Organization for Economic Co-operation and Development
OFMS: Oral and maxillofacial surgery
OMSB: Oman Medical Specialty Board
SDGs: Sustainable development goals
SPSS: Statistical Package for the Social Sciences
SQU: Sultan Qaboos University
WHO: World Health Organization
